# Recent advances in understanding the biological roles of the plant nuclear envelope

**DOI:** 10.1080/19491034.2020.1846836

**Published:** 2020-12-09

**Authors:** Norman Reid Groves, Alecia Biel, Morgan Moser, Tyler Mendes, Katelyn Amstutz, Iris Meier

**Affiliations:** a Department of Molecular Genetics, The Ohio State University, Columbus, OH, USA; b Center for Applied Plant Sciences, The Ohio State University, Columbus, OH, USA; c Center for RNA Biology, The Ohio State University, Columbus, OH, USA

**Keywords:** LINC complex, nuclear envelope, lamina, chromatin organization, stomata, pollen tubes, nodulation, calcium, virus

## Abstract

The functional organization of the plant nuclear envelope is gaining increasing attention through new connections made between nuclear envelope-associated proteins and important plant biological processes. Animal nuclear envelope proteins play roles in nuclear morphology, nuclear anchoring and movement, chromatin tethering and mechanical signaling. However, how these roles translate to functionality in a broader biological context is often not well understood. A surprising number of plant nuclear envelope-associated proteins are plant-unique, suggesting that separate functionalities evolved after the split of Opisthokonta and Streptophyta. Significant progress has now been made in discovering broader biological roles of plant nuclear envelope proteins, increasing the number of known plant nuclear envelope proteins, and connecting known proteins to chromatin organization, gene expression, and the regulation of nuclear calcium. The interaction of viruses with the plant nuclear envelope is another emerging theme. Here, we survey the recent developments in this still relatively new, yet rapidly advancing field.

## Introduction

The nucleus is enclosed by a double membrane called the nuclear envelope (NE), which consists of the outer nuclear membrane (ONM), the inner nuclear membrane (INM), the nuclear pore membrane, and the embedded nuclear pore complexes (NPCs; not reviewed here, for recent reviews see [1,2]). While each compartment is functionally distinct, they are all connected, and the proteins of the different substructures interact with one another. The INM and ONM are populated by a collection of functionally diverse integral or membrane-associated proteins. The ONM is a continuation of the endoplasmic reticulum, but harbors additional ONM-specific proteins, whereas the protein composition of the INM is determined by the requirement for the specific INM proteins to passage through the NPC (as reviewed in [3]).

**Table 1.** NE and NE-associated proteins in *Arabidopsis thaliana.*

**Gene Name**	**Accession Number**	**Protein Interactor(s)**	**Plant Phenotype**	**Cellular Phenotype**	**Subcellular Localization**	**References**
**Lamin-Like Proteins**						
CRWN1	At1g67230	SUN1/2, KAKU4, NTL9, PWO1	Dwarfed plant in *crwn1 crwn2, crwn1 crwn3, crwn1 crwn2 crwn4*, & *crwn1 crwn3 crwn4*; increased pathogen resistance in *crwn1, crwn1 crwn2*, and *crwn1 crwn4*; ABA hyposensitivity during seed germination in *crwn1* & *crwn1 crwn3*	Altered nuclear shape, Decreased nuclear size, altered nuclear organization	Nuclear periphery	31, 33, 34, 37, 55, 56, 59, 61, 64
CRWN2	At1g13220		Dwarfed plant in *crwn1 crwn2* & *crwn1 crwn2 crwn4*, moderate growth defect in *crwn2 crwn4*	Altered nuclear shape, altered nuclear organization	Nucleus	31, 33, 34, 61
CRWN3	At1g68790		Dwarfed plant in *crwn1 crwn3* & *crwn1 crwn3 crwn4*; ABA hyposensitivity during seed germination in *crwn3* & *crwn1 crwn3*	Altered nuclear shape, altered nuclear organization	Nucleus	33, 34, 55
CRWN4	At5g65770	KAKU4, PWO1, PUX3/4/5	Dwarfed plant in *crwn1 crwn2 crwn4* & *crwn1 crwn3 crwn4*, moderate growth defect in *crwn2 crwn4*	Altered nuclear shape, Decreased nuclear size, altered nuclear organization	Nuclear periphery	33, 34, 37, 99
KAKU4	At4g31430	CRWN1, CRWN4, PUX3/4/5	Male fertility defect	Altered nuclear shape, nuclear invaginations and deformations^GOF^	Nuclear periphery	37, 50, 99
**SUN Proteins**						
SUN1	At5g04990	WIP1/2/3, SINE1, SINE2, SINE3, SINE4, TIK, CRWN1, NEAP1/2/3, SUN3/4, PWO1, OPENER, PUX3/4/5	*sun1 sun2* double null is lethal; reduced seed set and male fertility defects in *sun1-KO sun2-KD*	Altered nuclear shape & nuclear positioning in roots; meiotic defects in *sun1-1 sun2-*2	INM	14, 15, 16, 18, 22, 23, 24, 33, 35, 36, 48, 64, 97, 99
SUN2	At3g10730	WIP1/2/3, SINE1, SINE2, SINE3, SINE4, CRWN1, NEAP1/2/3, SUN3/4, OPENER	*sun1 sun2* double null is lethal; reduced seed set and male fertility defects in *sun1-KO sun2-KD*	Altered nuclear shape & nuclear positioning in roots; meiotic defects in *sun1-1 sun2-2*	INM	14, 15, 16, 18, 22, 23, 24, 33, 35, 36, 48, 97
SUN3	At1g22882	SUN1, SUN2, SUN4, SUN5, WIP1, TIK	Triple mutant *sun3 sun4 sun5* is embryonic lethal		NE/ER	15
SUN4	At1g71360	SUN1, SUN2, SUN3, TIK	Triple mutant *sun3 sun4 sun5* is embryonic lethal	*sun4 sun5* has a nuclear size defect	NE/ER	15
SUN5	At4g24950	SUN3	Triple mutant *sun3 sun4 sun5* is embryonic lethal	*sun4 sun5* has a nuclear size defect	NE/ER	15
**KASH Proteins**						
SINE1	At1g54385	SUN1, SUN2, SINE2	Impaired stomatal dynamics	Nuclear positioning defect in guard cells	ONM	23, 39
SINE2	At3g30970	SUN1, SUN2, SINE1	Impaired stomatal dynamics		ONM	23, 39
SINE3	At3g06600	SUN1, SUN2			ONM	23
SINE4	At4g24950	SUN1, SUN2			ONM	23
WIP1	At4g26455	SUN1, SUN2, SUN3, WIT1, WIT2, RanGAP1/2, WPP1/2/3	Male fertility defect in *wip1 wip2 wip3*	Nuclear shape defects in roots, nuclear shape & movement defects in pollen in *wip1 wip2 wip3*	ONM	18, 19, 20, 22, 23
WIP2	At5g56210	SUN1, SUN2, WIT1, WIT2, RanGAP1/2, WPP1/2/3	Male fertility defect in *wip1 wip2 wip3*	Nuclear shape defects in roots, nuclear shape & movement defects in pollen in *wip1 wip2 wip3*	ONM	18, 19, 20, 22, 23
WIP3	At1g08290	SUN1, SUN2, WIT1, WIT2, RanGAP1/2, WPP1/2/3	Male fertility defect in *wip1 wip2 wip3*	Nuclear shape defects in roots, nuclear shape & movement defects in pollen in *wip1 wip2 wip3*	ONM	18, 19, 20, 22, 23
TIK	At5g44920	SUN1, SUN2, SUN3, SUN4	Decreased root length	Nuclear size defect	NE/ER	16
**NE-Associated Proteins**						
WIT1	At5g11390	WIP1/2/3, RanGAP1/2, WPP1/2/3, Myosin XI-i	Male fertility defect in *wit1 wit2*; reduced ROS sensitivity and altered nuclear calcium signatures in *wit1 wit2*	Nuclear shape defects in roots, nuclear shape & movement defects in pollen in *wit1 wit2*	ONM	18, 20, 21, 22, 23, 46
WIT2	At1g68910	WIP1/2/3, RanGAP1/2, WPP1/2/3, Myosin XI-i	Male fertility defect in *wit1 wit2*; reduced ROS sensitivity and altered nuclear calcium signatures in *wit1 wit2*	Nuclear shape defects in roots, nuclear shape & movement defects in pollen in *wit1 wit2*	ONM	18, 20, 21, 22, 23, 46
NEAP1	At3g05380	SUN1, SUN2, bZIP18		Increased nuclear size; reduction in heterochromatin in *neap1 neap 3*	INM	36
NEAP2	At5g26770	SUN1, SUN2, bZIP18			INM	36
NEAP3	At1g09470	SUN1, SUN2, bZIP18		Increased nuclear size; reduction in heterochromatin in *neap1 neap 3*	INM	36
DMI1	At5g49960	CNGC15	Root length and root development defects	Altered nuclear calcium signaling	NE	71
CNGC15	At2g28260	DMI1	Can functionally rescue *mtcngc15b,c* symbiosis defect		NE	71, 72
**Novel NE Proteins**						
OPENER	At5g43822	SUN1, SUN2	Embryonic Lethal	Nuclear/nucleolar size defect	NE	97
	At1g07970				NE	95
	At3g08870				NE	95
PNET1	At1g07970	CPR5, PNET6, Nup155, Nup58, Nup88	*pnet1 nup160* is embryonic lethal; Male fertility defect in *pnet1 hos1*		NE	96
PNET2	At5g67610				NE	96
PNET3	At2g46890				NE/ER	96
PNET4	At5g47400				NE	96
PNET5	At5g11560				NE/ER	96
PNET6	At2g39630	PNET1			NE	96
PNET7/MSBP1	At5g52240				NE	96
PNET8/HMG1	At1g76490				NE	96
PNET9/TMEM18	At1g34350				NE	96
PNET10	At5g48810				NE	96
PNET11	At3g60600				NE/ER	96
PNET12	At1g62810				NE	96
PNET13	At1g19370				NE	96
PNET14	At4g46280				NE/ER	96

Linker of nucleoskeleton and cytoskeleton (LINC) complexes are protein complexes embedded in the NE and form a direct connection between the cytoplasm and the nucleoplasm [4–7]. LINC complexes were first discovered in *Drosophila melanogaster* and *Caenorhabditis elegans* in mutant screens for defects in nuclear positioning and/or nuclear migration [8]. Crucial components of LINC complexes are two types of protein, INM-associated Sad1/UNC-84 (SUN) proteins and ONM-associated Klarsicht/ANC-1/Syne Homology (KASH) proteins. KASH proteins have a C-terminal, tail-anchored transmembrane domain and a short C-terminal tail that is bound in the NE lumen by the SUN domains of SUN protein trimers [9]. The canonical model is that SUN-KASH domain interactions across the NE connect the cytoskeleton with proteins of the nuclear interior.

Several examples of specific interactions between individual KASH proteins and the microtubule motors dynein and kinesin are known. Other KASH proteins interact directly or through adaptors with actin or intermediate filaments (reviewed in [7,10]). SUN proteins interact with nuclear lamins and chromatin-associated proteins in the nucleoplasm. LINC complex-cytoskeleton interactions are involved in nuclear movement and nuclear positioning in several developmentally important contexts. In this context, the LINC complexes have been proposed to act like nut, washer, and bolt in the NE to allow the force transmission required to move or anchor nuclei [4,11,12] Table 1.

**Figure 1.**
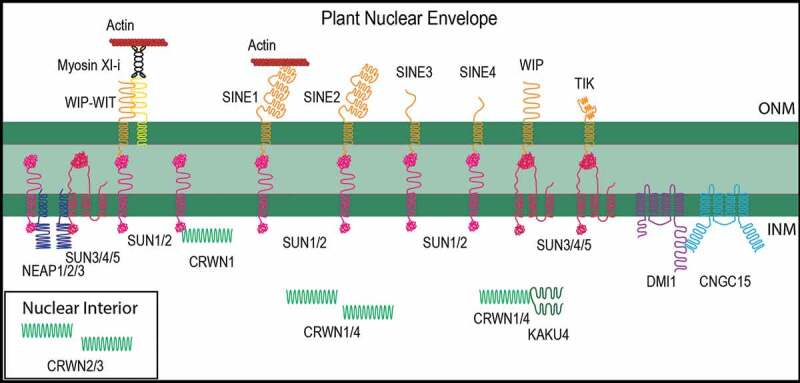
**Established Arabidopsis nuclear envelope-associated proteins and their interactions discussed here**. Outer nuclear membrane (ONM) proteins face the cytoplasm and interact with membrane-bound and cytoplasmic proteins. Plant KASH proteins (orange) reside at the ONM and interact directly or indirectly with the cytoskeleton (see WIP and SINE1 for examples). WIP (WIP1, WIP2 and WIP3) interacts with the tail-anchored membrane WIT (WIT1 and WIT2; yellow), which in turn interacts with Myosin XI–i. The inner nuclear membrane (INM) is populated by SUN proteins (pink), which come in two varieties: C-terminal SUNs (SUN1–2) and mid-SUNs (SUN3–5). C-terminal SUNs interact with KASH proteins in the NE lumen, as well as with NEAPs (blue), and the plant lamin CRWN1 (green) at the INM. Mid-SUNs interact with select KASH proteins, as well as NEAPs. The plant lamins CRWN1, CRWN4, and KAKU4 reside at the nuclear periphery, and CRWN1 interacts with KAKU4. The ion channels DMI1 and CNGC15 are located at the INM, and interact. For details, see text

Plant SUN proteins were first reported as *Arabidopsis* Sad1a and Sad1b [13] and later shown to be the plant homologs of animal SUN proteins [14,15]. Like in animals, there are two classes of plant SUN proteins, those that have the SUN-domain at the C-terminus (C-SUNs) and those with a centrally located SUN domain (Mid-SUNs). Unlike C-SUNs, Mid-SUNs are also located at the endoplasmic reticulum and are functionally less well understood [16,17] Figure 1.

While SUN proteins thus appear well conserved, no homologs of animal KASH proteins easily recognizable through sequence similarity searches can be found in plants. In Arabidopsis, the WPP domain-interacting proteins (WIPs) were the first identified plant analogs of animal KASH proteins, binding SUN proteins at the NE [18–21]. Together with their binding partners WPP domain-interacting tail-anchored proteins (WITs), they are involved in nuclear movement in Arabidopsis root hairs [18,22] and pollen tubes [23]. Additional Arabidopsis KASH proteins include Toll-Interleukin-Resistance KASH protein (TIK), which has a KASH tail more similar to animal KASH proteins [16], and SUN-interacting nuclear envelope proteins 1–4 (SINE1–4), which share a C-terminal VPT motif with WIP [24]. SINE1 and SINE2 are closely related and share an N-terminal armadillo-repeat (ARM) domain, while SINE3 and SINE4 are unrelated KASH proteins with short cytoplasmic domains of unknown function [24] Figure 1.

The animal INM is associated with an electron-dense layer of filamentous material named the nuclear lamina [4,25], which is composed of type V intermediate filaments called lamins [26–28]. Lamins are important nucleoskeleton components that can form direct or indirect contact with chromatin [29]. They bind SUN proteins and are linked to the cytoskeleton through LINC complexes, and are essential for nuclear shape, nuclear positioning, and mechanotransduction. Plants do not have a canonical nuclear lamina, although an electron-dense layer of unknown composition beneath the nuclear envelope has been reported [30]. A group of filamentous proteins at the nuclear periphery has been suggested to act as the structural and functional equivalent of lamins, known as plant-specific lamins or Plamins (as reviewed in [3]). Plamins were first discovered in *Daucus carota* nuclei (Nuclear Matrix Constituent Protein, NMCP1) and have since been identified in other plant species [30–32]. NMCP homologs in Arabidopsis are named CROWDED NUCLEI (CRWN) 1–4 (formerly known as LITTLE NUCLEI 1–4) [31,33,34]. CRWN1–4 share no significant amino acid similarity to lamins but they do share a tripartite structure and an extensive central coiled-coil domain. CRWN1 and CRWN4 are located at the nuclear periphery while CRWN2 and CRWN3 are located in the nuclear interior [31,34,35]. Another potential component of the plant nucleoskeleton is a family of proteins called NEAPs [36]. NEAPs, like CRWNs, are coiled-coil proteins; however, they are directly anchored to the NE by a transmembrane domain. They interact with both C-SUN and Mid-SUN proteins [36]. In addition, a novel NE-associated coiled-coil protein, KAKU4, has been identified. KAKU4 is located at the nuclear periphery and plays a role in nuclear shape determination, similar to CRWNs [37]. KAKU4 overexpression induces an increased production of nuclear membrane, which results in nuclear invaginations. KAKU4 interacts with CRWN1, and overexpression of CRWN1 also leads to an increase in nuclear membrane production, which results in nuclear deformations and invaginations (CRWNs and KAKU4 depicted in Figure 1).

Recently, the investigation of plant NE composition and function has gained momentum. Several new connections have been made to specific plant physiological and developmental pathways. New cellular and molecular roles of the NE proteins have been revealed, new proteins populating the NE have been discovered, and new methods have been developed or applied to the field. Here, we summarize novel findings of the past 3 years, illustrating the increasing dynamics and connectivity of the field and discussing its most pressing next questions.

## Role of LINC complexes in stomatal development and function

Higher plants have developed elaborate mechanisms to cope with their inability to escape changes in environmental stresses, such as drought, salinity, temperature, and pathogen invasion. Stomata are intricately regulated to respond to many of these stresses in order to maximize plant survival. Stomata are found predominately within the epidermal layer of leaves and are composed of two specialized cells known as guard cells creating a central pore through which gas exchange between the plant and its environment occurs. During drought or pathogen invasion, stomata can close, which conserves water loss and can prevent pathogen access (reviewed in [38]).

Recently, the Arabidopsis KASH proteins SINE1 and SINE2 were connected to the regulation of stomatal behavior [39]. In leaves, SINE1 is exclusively expressed in the guard cell lineage while SINE2 is expressed in epidermal cells, mesophyll cells, and trichomes, but only weakly in guard cells [24]. SINE1 is involved in guard cell nuclear positioning and co-localizes with F-actin, whereas SINE2 is involved in oomycete pathogen response and appears to have no role in stomatal nuclear positioning or F-actin interaction [24]. Interestingly, however, both SINE1 and SINE2 play an important role in stomatal opening and closing [39]. Loss of SINE1 or SINE2 results in ABA-hyposensitivity and increased drought susceptibility. The ABA-induced stomatal closure phenotype is, in part, attributed to impairments in Ca^2+^ and F-actin regulation, suggesting that SINE1 and SINE2 act downstream of ABA but upstream of Ca^2+^ and F-actin. SINE1 and SINE2 are also required for efficient stomatal opening and stomatal response to changes in light-dark conditions.

Despite many similarities, the mutants also differed in several points, such as the response to the actin stabilizing drug jasplakinolide or differential effects of overexpression, suggesting some functional divergence. To further unravel these differences, to investigate the role of SUN proteins and other LINC-complex associated factors, and to dissect the interactions between SINE1, SINE2 and the guard cell cytoskeleton should be focus areas of further studies.

Maize LINC KASH AtSINE-like2 (MLKS2) is the single *Zea mays* ortholog of Arabidopsis SINE1/2 [40]. MLKS2 has a C-terminal KASH tail, which is required for localization to the NE through binding of the KASH tail to maize SUN2 and an ARM domain that associates with F-actin [41]. Among the pleiotropic phenotypes of *Mlks2* mutants are defects in stomatal complex development. Subsidiary cells within the stomatal complex of maize *mlks2*–*1* and *mlks2*–*2* have severe defects such as size variance, shape abnormalities, and deviations from the normal ratio of two subsidiary cells per stoma. These phenotypes are suggested to be the result of cell polarization and nuclear migration impairments which were observed in the early stages of stomatal development [41]. The authors note that the phenotypes are similar to those of *pangloss* or *brick* mutants in maize, which are involved in nuclear positioning and regulation of the actin cytoskeleton [42,43]. How/if the loss of MLKS1 or MLKS2 affects stomatal function and actin organization remains to be tested.

Interestingly, the guard cell phenotype described for MLKS2 differs significantly from that discussed above for Arabidopsis SINE1 and SINE2. This might suggest sub-functionalization, or require further detailed studies into the role of the Arabidopsis proteins in guard cell development and the maize protein in guard cell physiology.

## Role of LINC complexes in male fertility

The WIP/WIT LINC complex plays a role in plant male fertility. Both protein families are associated with the nuclear envelope of the pollen nucleus (the vegetative nucleus, VN). During angiosperm pollen tube growth, the two sperm cells (SC) are linked to the surface of the VN through plasma membrane extension and the unit of nucleus plus sperm cells is called male germ unit (MGU) (as reviewed in [44]). As the pollen tube grows, the MGU maintains a fixed distance from the pollen tube tip, with the VN migrating ahead of the SCs [45]. *Wip* and *wit* mutant pollen tubes grow at normal rates, but the SCs precede the VN and distance of the VN to the pollen tube tip increases over time [23,46,47]. Frequently, the VN is not observed at the pollen tube tip, and those pollen tubes either stall at the micropyle or continue to grow past the synergid cells, consistent with a defect in pollen tube termination [23]. A *sun1 sun2* mutant also showed reduced seed set, reversed MGU order, and defects in male gametophyte development [46,48].

As *wip* and *wit* mutant pollen tubes frequently fail to burst, Moser et al. have now addressed whether the WIP/WIT LINC complex is required for reception of exogenous signals secreted from female tissue [49]. Loss of WIPs and/or WITs results in pollen tubes that are hyposensitive to exogenous H_2_O_2_, with reduced pollen tube rupture observed in mutant pollen tubes [47]. Treatment of WT and mutant pollen tubes with a Ca^2+^ channel inhibitor, GdCl_3_, prevented H_2_O_2_-induced rupture completely, suggesting that the reduced H_2_O_2_ response of the mutant is still Ca^2+^-dependent [47]. Sensitivity to exogenous H_2_O_2_ decreases over time, which coincides with decreased proximity of the VN to the pollen tube tip. This suggests that VN proximity to the pollen tube tip, mediated by the WIP/WIT LINC complex, is required for perception of H_2_O_2_, and potentially other exogenous signals.

Recently, KAKU4 and CRWN1 mutants have been analyzed for male fertility and MGU movement defects [50]. KAKU4 is highly expressed in Arabidopsis pollen and is predominantly located at the VN envelope. Loss of KAKU4 results in a slightly reduced seed set, more spherical nuclei, and a reversal in MGU order reminiscent of WIP and WIT mutants. Similar phenotypes were observed in a *CRWN1* mutant but are even less severe [50]. If, and how, KAKU4 and/or CRWN1 may be interacting (directly or indirectly) with the SUN-WIP-WIT complex to facilitate proper MGU movement and fertilization requires further investigation.

In *Zea mays*, mutants deficient in the SINE1/2 homolog MLKS2 exhibit defects in male fertility, pollen viability, and different stages of male meiosis. There had been prior evidence for an involvement of maize SUN proteins in meiosis [51]. The MLKS2 mutant now shows deviation from several cytological meiotic landmarks, in addition to reduced pollen viability, strengthening the hypotheses that maize LINC complexes are involved in telomere bouquet formation and suggesting that other plant LINC complexes of currently unknown function should also be assayed for male fertility defects [41].

## CRWN roles in plant-pathogen interactions and seed germination

CRWN proteins have recently been identified as playing a role in the regulation of pathogen defense signaling. Pathogen defense signaling is often regulated by cross talk between the salicylic acid (SA) and jasmonic acid (JA) pathways, two hormone pathways which are involved in pathogen defenses [52]. CRWNs are negative regulators of plant defense responses, and *crwn1, crwn1 crwn2*, and *crwn1 crwn4* mutants have increased pathogen resistance [53–55]. *Crwn1* mutants have increased pathogen resistance and an increased expression of JA and JA-associated genes, accompanied by a decreased expression of genes associated with pattern-triggered immunity (PTI) and genes regulated by SA [53].

*Crwn1 crwn2* and *crwn1 crwn4* mutants have increased basal levels of SA, as well as increased transcription of genes involved in the SA response, indicating that CRWN1, CRWN2, and CRWN4 in combination are involved in the SA pathway [54,55]. Several mechanisms for the regulation of the SA pathway involving CRWNs have been proposed. The CRWN1 C-terminus interacts with NAC WITH TRANSMEMBRANE MOTIF1-LIKE9 (NTL9), a transcription factor known to be involved in repressing *PATHOGENESIS-RELATED1* (*PR1*) [55]. The presence of CRWN1 increases the DNA-binding activities of NTL9, indicating CRWN1 plays a specific role in PR1 regulation [55]. In addition, Choi et al. propose a general role for CRWN1 in histone methylation [54]. The *PR1* promoter in *crwn1 crwn2* mutants has fewer repressive H3K27me3 marks [54], and CRWN1 and CRWN2 are required for H3K27me3 methylation of several loci including *PR1* [56], indicating that CRWNs may be involved in transcriptional regulation of biotic stress response proteins. An alternate model proposes that loss of CRWNs and subsequent nuclear shape changes [33] cause DNA damage, leading to SA signaling, which triggers the immune response. It has also been reported that CRWN1 degradation is induced by pathogen infection, indicating that plants may regulate the expression of pathogen defense genes by degrading the nuclear lamina [55].

Based on the known role of CRWNs in nuclear size control, recent studies have examined whether they are also involved in nuclear size changes during seed dormancy and seed germination [31,33]. Nuclear size decreases during seed dormancy and increases upon seed germination (reviewed in [57]). *Crwn1 crwn2* embryonic nuclei, already smaller than their WT counterparts, still decrease in size during seed maturation and dormancy and marginally increase during seed germination [58]. This nuclear size phenotype is counter to the *abi3* mutant, deficient in a positive regulator of the ABA pathway, in which nuclear size is increased both during seed dormancy and seed germination [57,58]. While these data indicate no direct role for CRWNs in regulating nuclear size during seed dormancy and seed germination, it is interesting that Zhao et al. report a role for CRWN1 and CRWN3 in the degradation of a positive regulator of the ABA pathway, ABI5 [59]. *Crwn1, crwn3*, and *crwn1 crwn3* are ABA hypersensitive during seed germination and *crwn1 crwn3* mutants accumulate higher levels of ABI5 during seed germination, when ABI5 is normally degraded in WT [59]. The C-terminal five amino acids of CRWN3 are required for this function, as a truncated form of CRWN3 is insufficient to rescue the ABA hypersensitivity in the *crwn1 crwn3* mutant [59]. While these data together suggest a direct role for CRWNs in regulating seed dormancy through the ABA pathway, further investigation will be required to determine the connection between the observed nuclear size changes and the effect on the ABA pathway.

Recent evidence has emerged that CRWN1 and CRWN3 also play a role in DNA damage repair. *crwn1 crwn3* seedlings have a decreased germination rate following exposure to the DNA damage agent methyl methanesulfonate (MMS) [60]. Treatment of *crwn* mutants with MMS also results in ROS hyperaccumulation, as well as increased DNA damage.

## Chromatin organization at the plant nuclear periphery and its connection to CRWN proteins

At the cellular level, CRWNs are most notably involved in maintaining nuclear architecture. Mutants that have lost all four CRWNs are non-viable and double and triple mutants have varying severity of phenotypes including reduced nuclear size and abnormal nuclear shape and heterochromatin organization [33]. CRWN1 and CRWN4 play a more significant role in nuclear architecture, as their single mutants have significantly decreased nuclear size and chromocenter number when compared to *crwn2* and *crwn3* single mutants [33]. Utilizing 3D-imaging techniques, the role of CRWNs in nuclear morphology and chromatin organization was investigated [61]. Nuclear volume, sphericity, and elongation as well as the distribution of chromocenters within the nuclear volume were significantly changed in a *crwn1 crwn2* double mutant. Chromocenters in WT nuclei were located close to the nuclear periphery, and this association was reduced in *crwn1 crwn2* [61].

Several studies on animal nuclei have shown that the transcriptional activity of a gene can be linked to its position within the three-dimensional nuclear space. Lamin-associated domains (LADs) have been described, which harbor repressed chromatin, and specific chromatin domains were mapped to the nuclear periphery. Whether a comparable chromatin organization exists in plants, and if so, which proteins of the nuclear periphery are involved in its establishment and maintenance is significantly less well established. Bi et al. have used the plant nucleoporin NUP1 fused to GFP in chromatin immunoprecipitation (ChIP) experiments to probe into the specific association of Arabidopsis chromatin domains with the nuclear pore and nuclear periphery [62]. They used restriction enzyme digests followed by a mild sonication to identify chromatin regions not preserved with classic ChIP methods [62]. Predominately pericentromeric regions were found associated with the nuclear periphery and these regions are enriched in heterochromatic marks [62].

The chromatin regions identified with NUP1 were used to design probes for fluorescent *in-situ* hybridization (FISH) to visualize chromatin tethering to the NE [63]. Indeed, originally identified NUP1-associated regions were more closely associated with the nuclear periphery than non-binding regions. Utilizing this experimental set-up on *crwn* mutants, it was shown that CRWN1 and CRWN4 are necessary for tethering specific chromatin regions to the nuclear periphery, with single mutants showing loss of chromatin enrichment at the NE and displaying overall less ordered chromatin compared to that of WT [63]. In addition, CRWN1 forms direct contacts with regions of repressive chromatin marks and lowly expressed genes and is enriched for the histone marker H3K27me3 [63].

Direct interaction between CRWNs, LINC complexes, and chromatin-regulatory proteins has also been found. CRWN1, CRWN4, and SUN1 physically and functionally interact with PWO1, a component of the repressive Polycomb-Group (PcG) complex, which associates with chromatin enriched in the repressive histone marker H3K27me3 [64]. PWO1 and CRWN1 proteins physically interact and influence the expression of an overlapping set of genes. Loss of PWO1 or CRWN1 reduced nuclear size to comparable levels, while *crwn1 pwo1* mutants restored the *crwn1* nuclear shape phenotype, indicating that CRWN1 and PWO1 may be acting within the same genetic pathway to influence nuclear size and shape. PWO1 is located in sub-nuclear foci associated with the nuclear periphery, suggesting that this could be the site of the PWO1–CRWN1 interaction.

In addition, Choi and Richards (2020) show that histone H3 lysine 27 tri-methylation levels are reduced in the absence of CRWNs near genes encoding transcription factors regulating SA biosynthesis, providing a possible explanation for the SA induction in CRWN mutants demonstrated by Choi et al. (2019) [54,56]. Together, the three studies suggest a connection between CRWNs and the repressive histone H3K27me3 mark, as well as between CRWNs and H3K27me3-associated proteins. CRWN proteins may therefore facilitate the tethering of repressed chromatin to the plant nuclear periphery or be actively involved in establishing or maintaining chromatin repression.

## Plant nuclear calcium signaling

Calcium (Ca^2+^) is a well-established and versatile secondary messenger that regulates a variety of cellular processes in both animals and plants [65,66]. Ca^2+^ signals are characterized by duration, frequency, and amplitude and vary in response to different stimuli (as reviewed in [67]). Additionally, spatial location of Ca^2+^ responses is tightly regulated in specific cell types and organelles in order to enable selective activation of downstream Ca^2+^-binding proteins and other targets [68–70].

Nuclear Ca^2+^ oscillations have been described in a variety of plant systems induced by various elicitors [70]. Leitão et al. showed that specific nuclear Ca^2+^ changes occur during growth in the root apical meristem and that this nuclear Ca^2+^ release is modulated by the Arabidopsis cation channel DOES NOT MAKE INFECTIONS 1 (DMI1) [71]. In *Medicago truncatula*, DMI1 localizes to the INM, interacts with cyclic nucleotide-gated channel 15 (CNGC15), and mediates the nuclear Ca^2+^ response to nodulation (Nod) or mycorrhizal (Myc) factors during root symbiosis Figure 1 [70,72–74]. In Arabidopsis, overexpression of DMI1 (*dmi1*–*2* mutant) leads to a decrease in nuclear Ca^2+^ spike frequency and shorter primary roots, suggesting overexpression of DMI1 may impair the activation or function of a nuclear Ca^2+^ channel. Knocking down DMI1 (*dmi1*–*1* mutant) results in longer roots and an increase in the length of time Ca^2+^ is released. However, no change in the nuclear Ca^2+^ spike frequency is observed. Addition of auxin is able to rescue the root length and nuclear Ca^2+^ defects observed in *dmi1*–*1* but not *dmi1*–*2*. This study suggests a new role for nuclear Ca^2+^ signaling in auxin signaling at the root apical meristem in Arabidopsis.

Ca^2+^ increases in both the cytoplasm and nucleus in plants has been demonstrated in several previous studies. However, the relationship between cytoplasmic and nuclear Ca^2+^ signaling is still not clear. Moser et al. describe pollen vegetative nuclear Ca^2+^ fluctuations both during pollen tube growth and in response to exogenous H_2_O_2_ [47]. When examining Ca^2+^ fluctuations in *wit1 wit2* pollen tubes, it was found that nuclear Ca^2+^ fluctuations, but not cytoplasmic Ca^2+^ oscillations, were impaired during growth and in response to H_2_O_2_ [47]. Those nuclear Ca^2+^ fluctuations were further reduced in pollen tubes where the VN was positioned further away from the pollen tube tip, suggesting that the proximity of the VN to the pollen tube tip is required for the establishment of the nuclear Ca^2+^ patterns [47]. Additionally, cytoplasmic and nuclear Ca^2+^ increases were observed after cold shock and NaCl treatment in Arabidopsis root epidermal cells [75]. The cytoplasmic and nuclear peaks were distinguished by the specific time delays, with cytoplasmic Ca^2+^ peak appearing first followed by the nuclear Ca^2+^ peak. Leitão et al. also observed time delays between cytoplasmic and nuclear Ca^2+^ signaling during root apical meristem growth [71]. Interestingly, the timing of the cytoplasmic and nuclear peaks during root growth was reversed, with the nuclear peak occurring first [71]. Both studies further suggest that cytoplasmic and nuclear Ca^2+^ responses can occur independent from each other.

Further investigation into the role of plant nuclear calcium in known cellular processes, such as auxin signaling, cold shock, hyperosmotic shock, and pollen tube growth and rupture, is needed. Additionally, this opens up the possibility that nuclear calcium may play a role in responses where cytoplasmic calcium signaling has been established.

## Plant viral interaction with the nuclear envelope

Vesicular transport between the cytoplasm and nucleus is becoming recognized as a cellular mechanism for the movement of cargoes across the NE [76]. While this phenomenon is mostly associated with nuclear viral egress in host cells, one study on *Wnt* signaling at the neuromuscular junction in *Drosophila* larvae found nuclear export of messenger ribonucleoprotein granules occurs through a similar transport pathway [77]. In this non-canonical pathway across the double membrane-bound nucleus, assembled virions are enveloped at the INM, pass through the perinuclear space, fuse with the ONM and are then delivered to the cytoplasm [76,78]. The study of vesicular transport in plants has mainly focused on viruses, but the mechanisms underlying this process are still poorly understood.

Nucleorhabdoviruses are a genera of negative-sense single-stranded RNA plant rhabdoviruses that infect monocot and eudicot plants and replicate and assemble within the nucleus [79]. The two most well-studied nucleorhabdoviruses are the sonchus yellow net virus (SYNV) and the potato yellow dwarf virus (PYDV). After entry into the cell, viral particles release nucleocapsid cores which are transported into the nucleus through NPCs [79]. Following primary transcription, nuclear export, and translation, viral proteins are imported back into the nucleus to participate in replication, formation of new nucleocapsid cores, and subsequent budding [79,80].

Plant rhabdoviruses have been shown to dramatically alter host-cell nuclear architecture to establish sites of viral replication and assembly [81,82]. All rhabdoviruses encode at least five proteins: nucleoprotein (N), phosphoprotein (P), matrix protein (M), transmembrane spike glycoprotein (G), and the RNA dependent RNA polymerase (L) [79]. Replication in the nucleus takes place in viroplasm of ring-shaped complexes composed of the viral mRNAs and N, P, and L proteins [82–84]. These sub-nuclear ring structures do not associate with nucleoli and the formation of the nucleocapsid cores occurs on the outer surface of the rings [84]. Infections also result in invagination of the INM into the nucleus and are believed to be required for virus budding [81,82,85]. Recently, Sun et al. demonstrated that this INM invagination is a result of interactions between the M protein and the membrane-anchored G protein [86]. Interestingly, INM invagination could be induced in uninfected cells by tethering the M protein to an endomembrane and co-expressing it with the C-terminal domain of the G protein or by artificially fusing the M protein to the G protein [86]. The NE-remodeling ability of the M protein is controlled by leucine residues 223 and 224 located within the nuclear localization signal (NLS), while the nuclear import function of the NLS is controlled by lysine and arginine residues 225 and 226. M proteins with mutations of the di-leucine were incapable of associating with the NE but were still imported into the nucleus, while mutations of the lysine and arginine led to the opposite result [87]. A putative nuclear export signal was found at the C-terminus of the M protein that may function in cell-to-cell movement; however, further characterization of this region is still required [87].

Additionally, M proteins have been shown to target NPCs to inhibit the export of host-cell mRNAs and function in super infection exclusion (SIE) [80,88]. SIE occurs when the primary virus infection prevents the infected cells from subsequent infection by the same or related virus. Deletion of the M protein, as well as mutating the NLS, resulted in the failure of the primary infection to inhibit transcription and super infection [80]. The current hypothesis is that M protein from the primary infection is either re-coiling or preventing the uncoiling of the nucleocapsids released by secondary invader, subsequently suppressing transcription and preventing super infection [80].

In summary, plant nucleorhabdoviruses can significantly alter host-cell nuclear architecture to allow for INM invagination and viral budding, as well as target NPCs to inhibit mRNA export. No studies have investigated whether the LINC complex and/or plant-specific lamins play a role in viral budding and maturation, thus opening up an exciting avenue of future experiments.

## Nuclear envelope protein conservation in the Streptophyta

Plant LINC complexes and LINC-complex associated proteins were originally identified in Arabidopsis, but other NE-associated proteins, e.g. of the NMCP family, have been described earlier in other species [89]. Early evidence for the existence of a physical structure resembling the animal nuclear lamina came from ultrastructural work in onions (reviewed in [30]). While in the post-genomic era, sequences for many homologs of the characterized proteins have been found and their phylogenetic relationships have been clarified, they have not been functionally investigated [18,24,90,91]. Recently, this has changed through the investigation of LINC complexes in two agriculturally important plant groups: legumes and grasses [40,41,92].

Poulet et al. performed a thorough phylogenetic analysis of nine NE-associated protein families [90]. The evolutionarily oldest protein family according to this analysis are the SUN proteins, conserved in Ostreococcus and Chlamydomonas, thus older than the split between the Chlorophyta and the Streptophyta. This is consistent with SUNs also being conserved across Metazoa and leaves room for the possibility that SUN proteins might have an ancestral function independent of their KASH counterparts. Of the plant-specific families, only CRWN and SINE1/2 are found in moss and Selaginella, while most other families are limited to the angiosperms and some proteins (SINE3, SINE4, TIK) even to a much narrower range, suggesting specialization of function. Ciska et al. found that the CRWN lineage is decidedly old, reaching back to the Charophyte algae, the basal Streptophyta, after branching of from the Chlorophyta, and thus older than the previously reported origin in moss [90]. Importantly, immunofluorescence microscopy demonstrated that the Physcomitrella CRWN homolog is located at the NE of protonema cells, thus suggesting functional conservation in early land plants [91].

On a narrower evolutionary scale, there are still vast distances between monocots and eudicots, in conjunction with significant biological differences. Given that monocots, and especially grasses, include some of the most important crop plants worldwide, investigation of NE proteins in monocots is relevant for both fundamental science and for their potential in crop improvement. Gumber et al. identified 22 homologs of plant NE-associated genes in *Zea mays*, including a new grass-specific plant KASH gene family [40]. They show that representative maize KASH proteins locate at the nuclear periphery and bind to maize SUN. Among the maize homologs are members of the SINE1/2, WIP, WIT, SUN, NEAP, CRWN, and KAKU4 families, suggesting overall broad conservation of the known NE proteome between monocot and eudicots.

As described above, MLKS2 is the single *Zea mays* homolog of Arabidopsis SINE1/2 [41]. Aside from its roles in stomatal development and pollen viability the MLKS2 mutant has a defect in root hair nuclear elongation. Heterologous overexpression experiments also suggest a role in nuclear anchoring, and a contribution to this role of the N-terminal ARM domain of MLKS2. Similar to Arabidopsis and Medicago SINE1 (see below), the ARM domain co-localizes with F-actin and the overexpressed protein seems to bridge F-actin and the ER. Surprisingly, maize MLKS2 expression rescues the nuclear shape phenotype of an Arabidopsis *wip1 wip2 wip3* triple mutant, suggesting that this phenotype of unknown biological relevance is not strictly diagnostic for the function of individual plant LINC complexes containing specific KASH proteins [41].

Within the eudicots, some plant groups are of particular interest for the investigation of a group of proteins involved in nuclear movement and nuclear anchoring. Observations since the 1950s have described targeted nuclear movement and positioning during symbiosis initiation between legumes and rhizobia and similar processes are observed for the accommodation of arbuscular mycorrhizal fungi (reviewed in [93]). Both symbioses, but in particular that between legumes and rhizobia leading to nitrogen fixation, are of vast agronomic importance. Understanding if LINC complexes are involved in these nuclear positioning events and if they are in turn important for symbiosis initiation is, therefore, a pressing question. Newman-Griffis et al. have identified homologs of Arabidopsis LINC complex protein families in the model legume *Medicago truncatula* [92]. These include homologs of WIP, WIT, SINE1, and SUN, as well as several new KASH proteins not conserved in the Brassicaceae. NE localization, dependence of KASH proteins on SUN protein binding for NE enrichment, and direct SUN-KASH binding are conserved for all Medicago proteins. Using overexpression of a SUN-derivative that accumulates at the ER (thus presumed to compete with endogenous SUN and deplete KASH proteins from the NE) they demonstrate that LINC complexes are necessary for proper nuclear shape and movement in *Medicago* root hairs, and that they are important for infection thread initiation and nodulation. The study does not yet address whether nuclear movement and positioning prior to and during infection thread formation depend on LINC complexes, and whether arbuscular mycorrhiza is equally affected. Identification of insertional mutants for individual Medicago KASH protein genes, while technically challenging, should now clarify these questions and add additional tools to the ongoing approaches to broaden the host range of nitrogen fixation in plants [94].

In summary, further functional investigation of this exciting group of proteins is now clearly warranted, both in economically important species and in those relevant for understanding evolutionary conservation and specialization of NE functions in the plant lineage.

## Expanding the nuclear envelope proteome

Two recent studies have focused on identifying novel plant NE proteins through proteomic screens. Goto et al. used sequential fractionation of isolated nuclei to increase the coverage in their proteomic screen [95]. Many previously identified nucleoporins and NE proteins were identified, validating the approach. Two new NE proteins of unknown function have been identified from this proteomic dataset thus far, At1g07970 and At3g08870 [95]. Tang et al. used two complementary approaches to identify new NE proteins [96]. First, a subtractive proteomic screen was conducted, which identified numerous nucleoporins and known NE proteins. From this screen, five novel NE proteins were identified, which were named plant nuclear envelope transmembrane proteins (PNET1–5). The second proteomic screen was conducted using proximity-labeling, which utilized known NE proteins and nucleoporins as baits in a BioID2 approach [96]. Two nucleoporins (Nup93a and Nup82), two KASH proteins (WIP1 and SINE1), and two INM proteins (SUN1 and NEAP1) were used as baits in this screen and ten additional PNETs were subsequently identified (PNET6–15).

PNET1 was shown through BiFC to interact with an array variety of nucleoporins, most strongly with the membrane nucleoporin CPR5, and the inner ring nucleoporin Nup155. A variety of nucleoporins were also identified using PNET1 as bait in the proximity-labeling proteomic screen, firmly establishing it as new plant nucleoporin. A *pnet1 nup160* double null is embryonic lethal, whereas a *pnet1 hos1* double mutant is sterile, an increase in severity compared to the *hos1* null mutant [96]. Further exploration of the NE protein candidates identified in these powerful proteomic screens and additional reiterative screening have the potential to rapidly identify new networks of functionalities at the NE.

Another INM protein of unknown function, OPENER, was recently identified [97]. OPENER is required for embryonic development and is named for a ‘pop cap’ (aka ‘ring pull’) cell morphology in *opnr* mutants prior to growth arrest, in which the nucleolus is enlarged, encompassing the majority of the nucleus. OPENER localizes to the NE and mitochondria, is a novel interactor of SUN1 and SUN2, and is dependent on SUN for NE interaction [97]. A partial rescue of the *opnr* mutant results in enlarged nuclei in root cells, indicating a potential role for OPENER in nucleolar and nuclear size control [97].

The BioID2 approach described above is also shedding first light on INM-specific protein degradation in plants. Among the interactors of SUN1, Huang et al. identified CDC48, a chaperone involved in extracting membrane proteins for hydrolysis by the 26S proteasome, its cofactors UFD1 and NPL4, as well as a group of plant ubiquitin regulatory X domain-containing proteins (PUXs) [98]. Five members of the CDC48 complex were identified as interactors of the INM proteins SUN1 and NEAP1: PUX3, PUX4, PUX5, UFD1B, and UFD1C. PUX3–5 were additionally shown to interact with CRWN4 and KAKU4. Treatment with a proteasome inhibitor, MG132, resulted in an increase in SUN1 accumulation, confirming SUN1 is a target of proteasome degradation [98]. Loss-of-function mutants for PUX3–5 resulted in an increase in SUN1 protein, suggesting PUX3–5 negatively regulates SUN1 degradation [98]. It remains to be determined if PUX3–5 interface with and regulate INM proteins generally, or SUN proteins specifically and what role the interaction with CRWN4 and KAKU4 plays in this process.

Transmembrane proteins embedded in the IMN have to be transported to their final location by passage through the NPC. In animals, several pathways for membrane protein trafficking to the INM have been identified, most notably the diffusion-retention model and the transport-factor-mediated model [76]. A hypothesized 60kDa size barrier for nucleocytoplasmic domains has been proposed in animals. None of these pathways to the INM had been demonstrated in plants. In *N. benthamiana*, fusion of an NLS to the Arabidopsis tail-anchored ER protein PICL is sufficient for enrichment at the NE, as well as localization to the INM [99]. A chimeric tail-anchored membrane protein, containing an NLS, intrinsically disordered domain, and transmembrane domain was sufficient to traffic to the INM. A variety of NLSs were sufficient for trafficking to the INM, including five different classes of monopartite NLS, as well as bipartite NLSs. A nucleocytoplasmic domain of over 60 kDa was tolerated for INM trafficking [99]. This study provides evidence for similar pathways to the INM existing in plants as are present in animals. Therefore, membrane proteins containing an NLS in their nucleocytoplasmic domain may be candidates to reside at the INM.

## Highlights and Outlook

- Arabidopsis KASH proteins SINE1 and SINE2 are involved in the stomatal response to light, dark, ABA, and diurnal cycles, in part attributed to impairments in Ca^2+^ and actin regulation.
- The maize SINE1 homolog MLKS2 is involved in stomatal complex development, male meiosis, pollen viability, root development, and nuclear morphology.
- The Arabidopsis WIT-WIP-SUN LINC complex is involved in male fertility, movement of the pollen nucleus, pollen tube ROS sensitivity, and pollen tube nuclear calcium fluctuations.
- The Arabidopsis lamin-like proteins KAKU4 and CRWN1 are involved in male fertility and positioning of the pollen nucleus.
- Medicago LINC complexes play a role in rhizobial infection thread formation and the initiation of nodulation.
- CRWNs are involved in pathogen defense signaling, involving both the SA and JA pathways.
- CRWNs are involved in seed dormancy and seed germination, possibly through transcriptional regulation.
- CRWNs are involved in chromatin organization, Histone H3K27 trimethylation and the association of specific chromatin regions with the nuclear periphery.
- CRWN1, CRWN4 and SUN1 physically and functionally interact with PWO1, a component of the repressive Polycomb-Group (PcG) complex.
- Nuclear calcium spiking is involved in auxin-based root elongation.
- Plant nucleorhabdoviruses alter host-cell nuclear architecture, leading to specific INM remodeling associated with viral budding.
- While some plant NE-associated proteins are clade-specific, many are deeply conserved within plants, reaching back to moss or even to the Charophyte algae, the most basal Streptophyta.
- Sequential fractionation, subtractive proteomics, and BioID2-based proximity labeling have provided a significant number of new plant NE proteins, including a new nucleoporin and components of an INM-associated protein degradation pathway.
- OPENER is a new NE protein that binds SUN1 and SUN2 and is required for embryo development and nuclear and nucleolar size control.
- An NLS-dependent pathway for nuclear import and INM association of tail-anchored proteins in plants has been discovered.

As seen by these highlights, the field of plant nuclear envelope biology is rapidly progressing to assign important biological roles to individual proteins and protein complexes, to discover more, and more plant-unique, nuclear peripheral proteins, to gain first insights into their roles in chromatin architecture, to apply new methods to the field, and to expand the horizon from the highly useful Arabidopsis model system to both important crop plants and a broader evolutionary context within the Streptophyta. Numerous routes for further fruitful research have now been paved, which will likely lead to a better understanding of functional interaction networks at the plant NE, to connecting the newly discovered biological roles back to the molecular roles of the proteins, to comparative functional studies between different plant species, and to approaches addressing the connection between the cytoplasmic and nuclear activities of the NE spanning complexes. Plant viruses interacting with the nuclear envelope can become a tool not only for their own understanding but for that of nuclear envelope biology, as well. Further down the road, and with much of this in place, the field can return to the overarching question of why and how Streptophyta and Opisthokonta have populated their nuclear envelopes with such diverse proteomes, and what this means for the emergence of both the components and their biological roles on a deep evolutionary time scale.
